# Exploration of the Potential Mechanism of Qi Yin San Liang San Decoction in the Treatment of EGFRI-Related Adverse Skin Reactions Using Network Pharmacology and *In Vitro* Experiments

**DOI:** 10.3389/fonc.2022.790713

**Published:** 2022-03-15

**Authors:** Yalei Wang, Yali Zhang, Chengcheng Ding, Caixia Jia, Huawei Zhang, Tiantian Peng, Shuo Cheng, Weihang Chen, Yan Tan, Xu Wang, Zhaoheng Liu, Peng Wei, Xue Wang, Miao Jiang, Qian Hua

**Affiliations:** ^1^ School of Tradition Chinese Medicine, Beijing University of Chinese Medicine, Beijing, China; ^2^ School of Life Scienses, Beijing University of Chinese Medicine, Beijing, China; ^3^ School of Acupuncture-moxibustion and Tuina, Beijing University of Chinese Medicine, Beijing, China; ^4^ Department of Pharmacy, Beijing Tsinghua Changgung Hospital, School of Clinical Medicine, Tsinghua University, Beijing, China

**Keywords:** QYSLS, EGFR inhibitor, skin adverse reaction, network pharmacology, traditional Chinese medicine

## Abstract

**Background:**

Adverse skin reactions are the most common side effects of epidermal growth factor receptor inhibitors (EGFRIs) in the treatment of cancer, significantly affecting the survival rate and quality of life of patients. Qi Yin San Liang San Decoction (QYSLS) comes from folk prescription and is currently used in the clinical treatment of adverse skin reactions caused by EGFRIs. However, its therapeutic mechanism remains unclear.

**Objectives:**

To explore the potential mechanism of QYSLS in the treatment of adverse skin reactions caused by EGFR inhibition using network pharmacology and experimental research.

**Methods:**

First, we verified the effectiveness of QYSLS *in vivo* using model mice. Second, the related targets of adverse skin reactions associated with EGFR inhibition were predicted by the Gene Expression Omnibus (GEO) database, and effective components and predictive targets of QYSLS were analyzed by Traditional Chinese Medicine Systems Pharmacology (TCMSP) and Batman-TCM databases. Gene ontology and Kyoto Encyclopedia of Genes and Genomes pathway analyses were performed *via* the Bioconductor (R) V3.8 bioinformatics software. Molecular docking studies verified the selected key ingredients and targets. Finally, the results of network pharmacology were verified by *in vitro* experiments.

**Results:**

In the *in vivo* mouse model, QYSLS effectively reduced the occurrence of skin side effects. Network pharmacological results showed that the active ingredient luteolin, quercetin, licochalcone a, and kaempferol and the effective targets prostaglandin-endoperoxide synthase 2 (PTGS2), matrix metallopeptidase 9 (MMP9), and C–C motif chemokine ligand 2 (CCL2) were related to the interleukin-17 (IL-17) and tumor necrosis factor (TNF) pathway. Subsequently, the related active compounds and targets were verified using HaCaT cells as an *in vitro* adverse reaction model. The results showed that luteolin and quercetin increased the expression of PTGS2 and MMP9 and reduced the expression of CCL2 in HaCaT cells treated with gefitinib.

**Conclusions:**

The results revealed that QYSLS effectively treats EGFRI-related adverse skin reactions through multi-target and multi-pathway mechanisms. Luteolin and quercetin may be the core active ingredients of QYSLS in the treatment of EGFRI-related adverse skin reactions, and their therapeutic effects are potentially mediated through PTGS2, CCL2, and MMP9 in the IL-17 and TNF signaling pathway.

## Introduction

According to the global cancer incidence and mortality data released by The International Agency for Research on Cancer in 2020, the incidence of lung cancer (11.4% of all cancer types) ranks second worldwide, and the mortality of lung cancer ranks first globally (18.0% of all cancer deaths) (1). Case studies have found that epidermal growth factor receptor (*EGFR*) gene mutation accounts for approximately 70% of the pathogenesis of non-small cell lung cancer in China (2, 3), representing the most common mutation type. EGFR inhibitors (EGFRIs) target non-small cell lung cancer caused by mutations in the *EGFR* gene. For example, the first-generation targeted drug gefitinib was approved to be imported to China in 2005 for the oral treatment of locally advanced and metastatic non-small cell lung cancer.

Clinical observation has found that EGFRIs are often accompanied by rash and other adverse reactions, and some patients are even forced to discontinue targeted drug therapy in severe cases. In lung cancer treatment with EGFRIs, common adverse reactions include rash, alopecia, abdominal pain and diarrhea, paronychia, and pulmonary fibrosis, with the highest incidence of rash up to 60.2% (3). Rash is closely related to gefitinib and generally occurs within 1–2 weeks after treatment, with the peak observed at 4–6 weeks followed by severe skin changes at 6–8 weeks (4). Clinically, when moderate to severe rash occurs, patients are usually complicated with local or even systemic infection. Approximately 76% of patients with rash and infection report negative effects on their quality of life, and 32% experience interrupted treatment due to intolerance (5). Although the second and third generations of targeted drugs are better tolerated, such as afatinib and osimertinib, many patients still present with rash symptoms (6). In addition, there are limited treatments for rashes caused by targeted drugs. At present, treatment plans for EGFRI-associated rash have been developed internationally, mainly including local and systematic treatment. Locally, Vaseline or hormone ointments are applied externally, and antibiotics (e.g., minocycline hydrochloride) or antihistamines (e.g., diphenhydramine) are used for systematic treatment (7). However, long-term external use of hormone drugs often leads to increased skin sensitivity and tenderness, accompanied by various symptoms, such as dryness, chafing, pigmentation, and even secondary infection in severe cases. Furthermore, the beneficial effects of oral antibiotics or antihistamines on improving the patients’ rash are limited, and they may cause liver and kidney toxicity, affecting the patients’ quality of life and potentially aggravating their condition (8). Therefore, finding intervention strategies that alleviate side effects (such as rash) is essential to improving patients’ quality of life and ensure effective treatment.

Qi Yin San Liang San Decoction (QYSLS) obtained from folk prescription has been widely used in clinical practice and is composed of 30 g of raw *Astragalus membranaceus*, 30 g of *Lonicera japonica*, 30 g of *Angelica sinensis*, 10 g of raw licorice, and 1 g of centipede. It has beneficial effects on qi and blood, heat clearing, and detoxification and is clinically used to treat EGFRI-associated acne-like rashes. However, the substance basis and mechanism involved in the treatment of EGFRI-induced dermal adverse reactions with QYSLS remain unknown.

In most cases, traditional Chinese medicine (TCM) achieves therapeutic effects by targeting multiple physiological pathways (9). Therefore, it is necessary to explore new perspectives and novel ideas to accurately elucidate the mechanism of TCM compounds. After years of investigation, gene regulatory network and network pharmacology theories have been established. In recent years, network pharmacology has been applied to study the therapeutic effects and targets of TCM and bioactive compounds. Network pharmacology is an emerging discipline in the era of big data that integrates systems biology, molecular biology, pharmacology, and a variety of network-computing platforms. It conducts multi-level network screening and construction from macro to micro perspectives and uses several database platforms and computer software programs to visualize data. Network pharmacology provides a more direct explanation of correlations between TCM compounds and diseases, including the molecular mechanisms underlying multi-molecule, multi-target, and multi-pathway interactions (10). This research model reflects the advantages of TCM compounds in the overall regulation of the body and can be used to reveal the effective components and molecular mechanism of QYSLS in the treatment of adverse skin reactions caused by EGFRIs.

**Figure 1:**
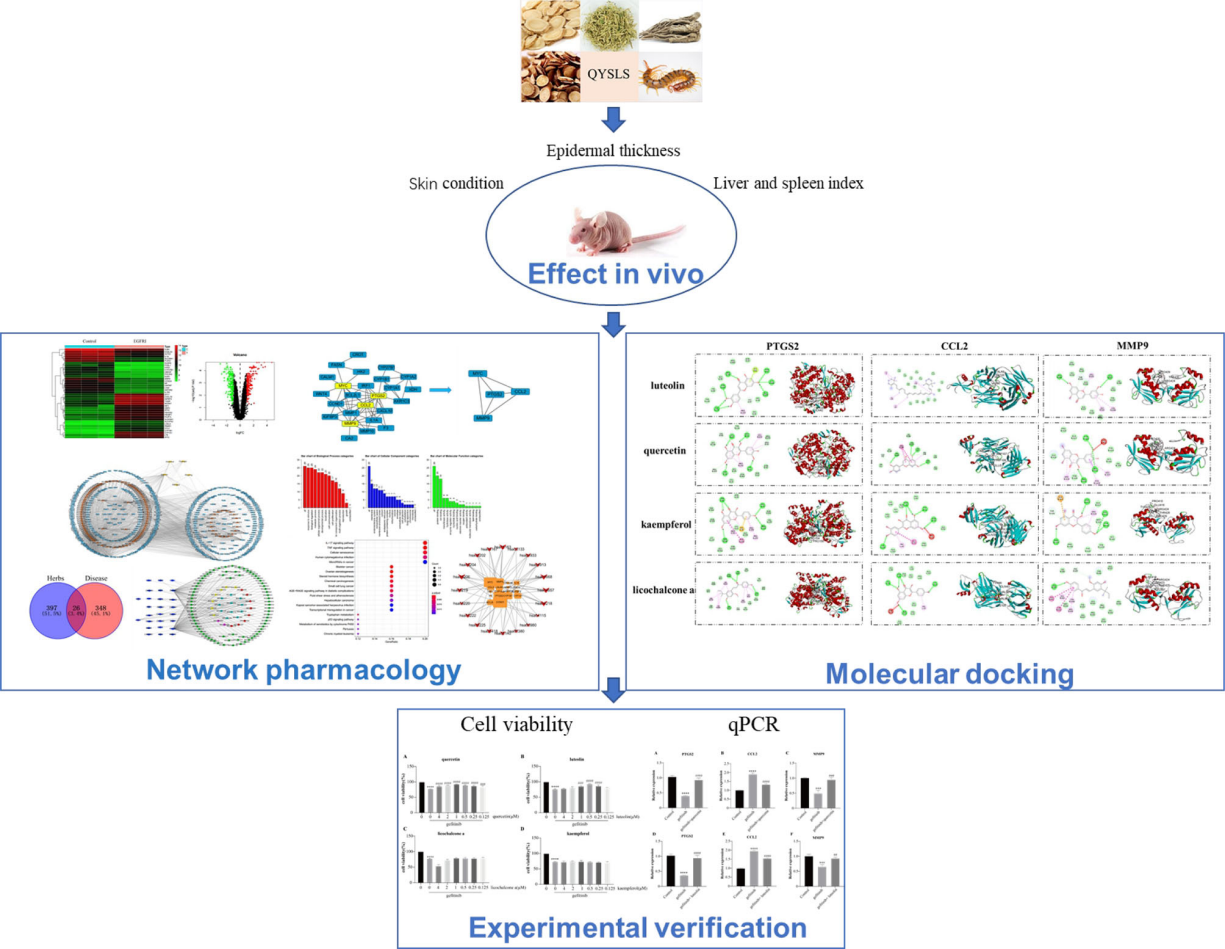
Flowchart of the network pharmacology and experimental study of QYSLS in the treatment of adverse skin reactions caused by EGFR inhibition.

In this study, a mouse model of adverse skin reactions was established to verify the clinical effectiveness of QYSLS in the treatment of skin-related side effects caused by EGFR inhibition. Furthermore, its potential molecular mechanism was studied through network pharmacology and molecular docking technology. Finally, its effective chemical components and molecular targets were verified through *in vitro* cell experiments. The schematic diagram is shown in **Figure 1**.

## Materials and Methods

### *In Vivo* Experiments

#### Chemicals and Drugs

Gefitinib (purity ≥ 98%) was purchased from Ark Pharm (Arlington Heights, IL, USA, art. No. Ak-72948), with a molecular weight of 446.90. All 5 types of Chinese medicines of QYSLS were purchased from Beijing Yizhentang Chinese Medicine Clinic Co., Ltd. Specifically, 1.3 l water was added to 90 g *Astragalus membranaceus*, 90 g *Lonicera japonica*, 90 g *Angelica sinensis*, 30 g licorice, and 3 g centipede and decocted for 45 min. For the second decoction, 0.8 l water was added and decocted for 30 min. Finally, the filtrate was combined and concentrated until the crude drug concentration was 3.03 g/ml (11, 12).

#### Experimental Animals

Male BALB/C NU/NU nude mice aged 4–5 weeks were used. After 1 week of adaptive feeding, the mice were divided into 5 groups, including the blank group, gefitinib group, gefitinib+ QYSLS low-dose group (15.15 g/kg, equal clinical dose conversion), gefitinib+QYSLS middle-dose group (30.3 g/kg, double clinical dose conversion), and gefitinib+QYSLS high-dose group (60.6 g/kg, quadruple clinical dose conversion). Gefitinib was given at 9 a.m. and QYSLS at 3 p.m (11).. The blank group was given the same dose of solvent. The drug was administered by gavage. After 14 days of feeding, the mice were anesthetized, and their skin conditions were recorded and photographed. After the mice were sacrificed, the skin of each mouse was collected for hematoxylin and eosin staining, and the liver and spleen were weighed to calculate the liver index and spleen index. All experimental procedures were approved by the Animal Ethics Committee of Beijing University of Chinese Medicine (ethical approval number: BUCM-2016103101-1008).

### Network Pharmacology Study

#### Collection of Targets Related to Adverse Skin Reactions Induced by EGFR Inhibition

Using “EGFRI skin normal” as keywords in the National Center for Biotechnology Information Gene Expression Omnibus (GEO) database (13) (https://www.ncbi.nlm.nih.gov/gds), samples were retrieved, and the GSE74407 gene expression profile related to EGFRI adverse skin reactions was downloaded. Nine samples were collected, including 3 normal human keratinocyte cell lines, 3 human keratinocyte cell lines treated with TNFα, and 3 human keratinocyte cell lines treated with TNFα and EGFRIs. Using the R language program to analyze two sets of samples and adjusted p-values < 0.05, differentially expressed genes (| logFC | ≥ 2) were screened.

#### Constructing the Database of Candidate Compounds

The Traditional Chinese Medicine Database and Analysis Platform (14) (TCMSP, http://tcmspw.com/tcmsp.php) was used to determine the effective components in QYSLS. This platform is a comprehensive database of natural component targets in China, integrating pharmacokinetics, pharmacochemistry, and drug-target protein-disease networks. In addition, the Batman-TCM database (http://bionet.ncpsb.org/batman-tcm/) was used to collect the active ingredient of centipede, and finally with OB 30% or higher and DL acuity 0.18 for the standard screening of 5 types of Chinese native medicine ingredient targets.

#### Generating the Protein–Protein interaction Network

The intersection targets found in STRING (15)(Version 10.5, https://string-db.org/) were analyzed to investigate protein–protein interactions (PPIs). Network nodes and edges represent proteins and protein–protein binding interactions, respectively. The PPI interactive network was constructed, and Cytoscape software (version 3.6.0) was used for visualization. Two times the average degree of freedom was selected as the minimum degree value under the Select option in Cytoscape software, and the core targets were screened to identify the core PPI network.

#### Gene Ontology and Kyoto Encyclopedia of Genes and Genomes Pathway Enrichment Analysis

Gene ontology (GO) analysis and Kyoto Encyclopedia of Genes and Genomes (KEGG) pathway enrichment were performed using Bioconductor (R) V3.8 bioinformatics software (http://bioconductor.org/). Pathway enrichment analysis was performed using the KEGG database to verify the functional classes of statistically significant genes (p < 0.05). The filter count threshold was ≥ 2, and terms with a systemic explorer score ≤ 0.05 were collected for functional annotation clustering.

#### Validation of Compound–Target Interactions

Crystal structures of hub protein targets were obtained from the RCSB PDB database (PDB, https://www.rcsb.org/). The MOL2 format structures of candidate active compounds were downloaded from TCMSP. Before docking, energy minimization was used for ligands and acceptors, water molecules were removed from acceptors (PDB files), polar hydrogen atoms were added, charge and magnetic field were provided, and then the files were saved in the PDBQT format using AutoDockTools (16) (version 1.5.6, http://autodock.scripps.edu/). The AutoDock platform was used for molecular docking verification, and all parameters were set to the default. The binding energy was calculated to evaluate binding interactions between the compounds and their targets. A binding energy less than −5 indicates a good binding interaction between the compound and target. The results were visualized by DS software, and the hydrogen bonds and their binding sites were observed and analyzed.

### *In Vitro* Experiments

#### Chemicals and Drugs

Luteolin, quercetin, licochalcone a, and kaempferol (purity ≥ 98%) were purchased from Shanghai Yuanye Biotechnology Co., Ltd. HaCaT was purchased from Dingguo Changsheng Biotechnology Co., Ltd., and it has been identified.

#### Cell Culture and Cell Viability Measurements

A Cell Counting Kit-8 solution (CCK-8; Dojindo, Rockville, MD, USA, CK04) was used to evaluate the effects of luteolin, quercetin, licochalcone a, and kaempferol on HaCaT cells. HaCaT cells were inoculated into 96-well plates at a density of 6 × 10^3^ cells/100 µl and treated with different concentrations of gefitinib, luteolin, quercetin, licochalcone a, and kaempferol for 24 h. The cells were then incubated with 10 µl CCK-8 solution for 2 h. The optical density at 450 nm was determined.

#### qPCR Assay

After a sufficient amount of cells was collected, the supernatant was removed and washed with phosphate-buffered saline. Total RNA was extracted using an RNA extraction kit (DP451). An ultraviolet spectrophotometer was used to assess RNA purity and concentration. The extracted RNA was reverse transcribed into cDNA. The cDNA was used as the template and amplified following the qPCR instructions (LABLEAD, R0202). GAPDH was used as an internal reference to calculate the relative expression of each target mRNA.

### Statistical Analysis

The results were analyzed using GraphPad Prism 8.0.2 software. The data were expressed as the mean ± SD. One-way analysis of variance was performed to compare the between-group quantitative data, and p < 0.05 indicated a significant difference.

## Results

### *In Vivo* Experiments

#### Effect of QYSLS on Cutaneous Adverse Reactions Induced by Gefitinib

**Figure 2:**
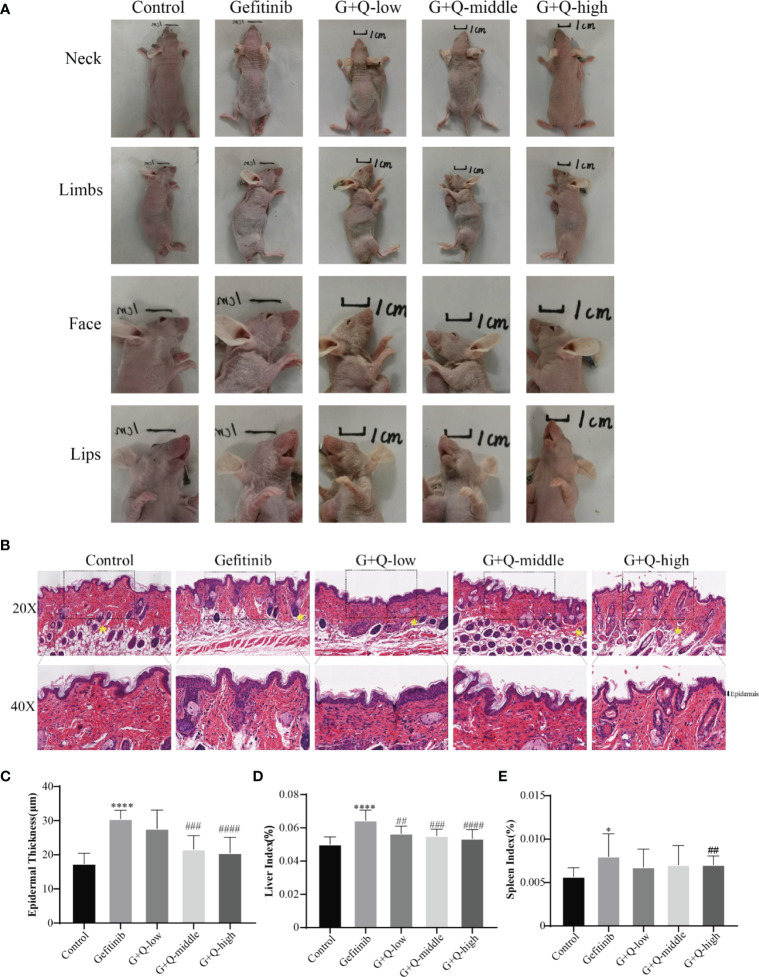
*In vivo* experiments showed that QYSLS effectively improved the adverse skin reactions caused by gefitinib. **(A)** Skin condition of the neck, limbs, face, and lip of mice in each group. **(B)** H&E staining of skin tissue from mice in each group. **(C)** Quantitative analysis of skin epidermal thickness. **(D)** Liver index of mice in each group. **(E)** Spleen index of mice in each group. “*” compared with Control, *p < 0.05, ****p < 0.0001. “^#^” compared with Gefitinib, ^##^p < 0.01, ^###^p < 0.005, ^####^p < 0.0001.

QYSLS has been successfully used for the clinical treatment of skin rashes caused by EGFRIs. We used an EGFRI-induced adverse skin reaction model to study the effect of QYSLS in mice. We established a cutaneous adverse reaction model by administering 225 mg/kg gefitinib for 14 days. The low-dose, middle-dose, and high-dose QYSLS groups received 15.15, 30.3, and 60.6 g/kg, respectively. The results showed that gefitinib induced adverse skin reactions in mice, mainly characterized by redness, desquamation of the neck, face, and limbs, and lip swelling. Treatment with different concentrations of QYSLS alleviated the adverse skin reactions caused by gefitinib (**Figure 2A**). Hematoxylin and eosin staining showed that QYSLS treatment significantly reduced epidermal thickening induced by gefitinib. In addition, QYSLS treatment improved fat deposition (**Figures 2B, C**) and ameliorated the abnormal increases in liver and spleen indices caused by gefitinib (**Figures 2D, E**). Together, these findings indicate that QYSLS reversed the immune damage caused by gefitinib and played a protective role. The above *in vivo* experimental data demonstrate that QYSLS effectively treated adverse skin reactions and immune damage caused by gefitinib.

### Network Pharmacology Study

#### Genes Related to EGFRI-Induced Adverse Skin Reactions

**Figure 3:**
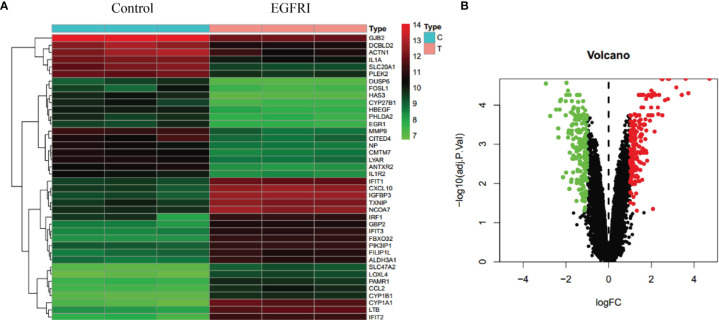
Collection of EGFRI-related adverse skin reaction targets. **(A)** Heat map of the top 40 differentially expressed genes. **(B)** Volcano map of the top 40 differentially expressed genes.

To predict the molecular mechanisms by which drugs exert their pharmacodynamic effects, we collected targets for EGFRI-related adverse skin reactions from publicly available microarray data. The search keyword was “EGFRI skin normal.” We downloaded GSE74407 data from the GEO database. In these data, Cavani et al. compared genetic changes in keratinocytes before and after EGFRI treatment (17). The adjusted p-value and logFC were used to analyze the chip data. An adjusted p-value < 0.05, log FC > 1, or log FC < −1 were the screening conditions for significantly different genes. A total of 374 differentially expressed genes were identified from the microarray data of the GEO repository, including 185 upregulated genes and 189 downregulated genes. The results are shown in **Figure 3**.

#### QYSLS Active Ingredients

**Figure 4:**
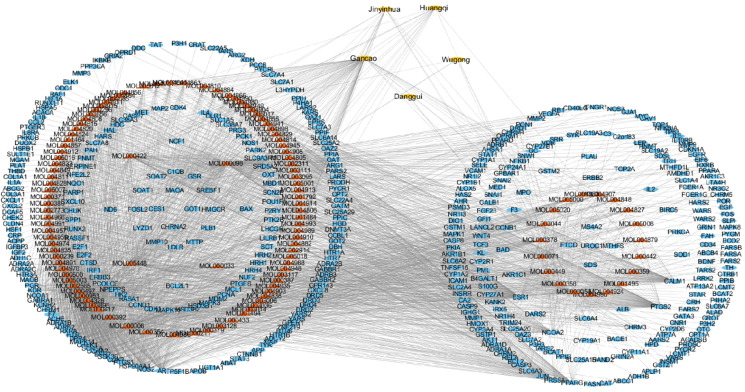
Ingredient-target network of QYSLS.

QYSLS is composed of *Astragalus membranaceus*, *Lonicera japonica*, *Angelica sinensis*, licorice, and centipede. In this analysis, 117 compound nodes were screened based on OB ≥ 30% and DL ≥ 0.18. The compound target network was composed of 545 nodes and 5,120 edges, including 5 TCM component nodes, 117 compound nodes, and 423 target nodes. Therefore, we speculate that the active compounds of QYSLS may affect multiple targets to effectively treat EGFRI-related adverse skin reactions. In this network, the relationship between the active compounds of QYSLS and their targets and potential pharmacological effects were directly explained (**Figure 4**).

#### Intersection of Drug Target Genes and Disease Target Genes

**Figure 5:**
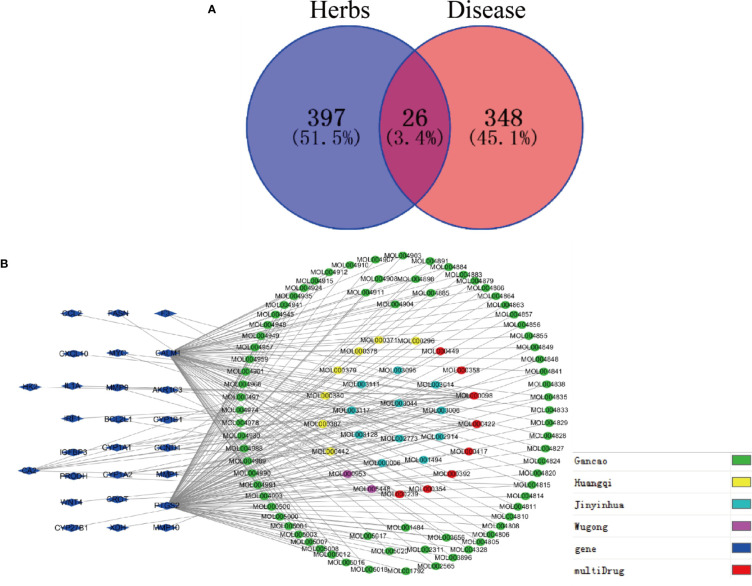
Screening of QYSLS-related compounds and construction of component target network. **(A)** Venn diagram of targets of herbs and diseases. **(B)** The compound–compound target network for QYSLS in treating EGFRI-related adverse skin reactions.

Next, Venny 2.1 was used to draw a Venn diagram of proteins, and **Figure 5A** was prepared after optimization. There were 26 proteins with common targets of components and diseases. The compound target network diagram for the treatment of EGFRI-related adverse skin reactions with QYSLS was further constructed, consisting of 129 nodes and 216 edges, including 123 compound nodes and 26 target nodes (**Figure 5B**). The main active substances were quercetin, kaempferol, beta-carotene, luteolin, and licochalcone a, which were screened by degree (see **Table 1** for details).

**Table 1:** Network node characteristic parameters of main active ingredients of QYSLS.

MOL ID	The active ingredient	Degree	Betweenness centrality	Closeness centrality
MOL000098	Quercetin	16	0.14812009	0.49799197
MOL000422	Kaempferol	7	0.09127254	0.47509579
MOL000006	Luteolin	5	0.02057035	0.45756458
MOL000497	Licochalcone a	4	0.01706673	0.46096654

#### PPI Network Analysis

**Figure 6:**
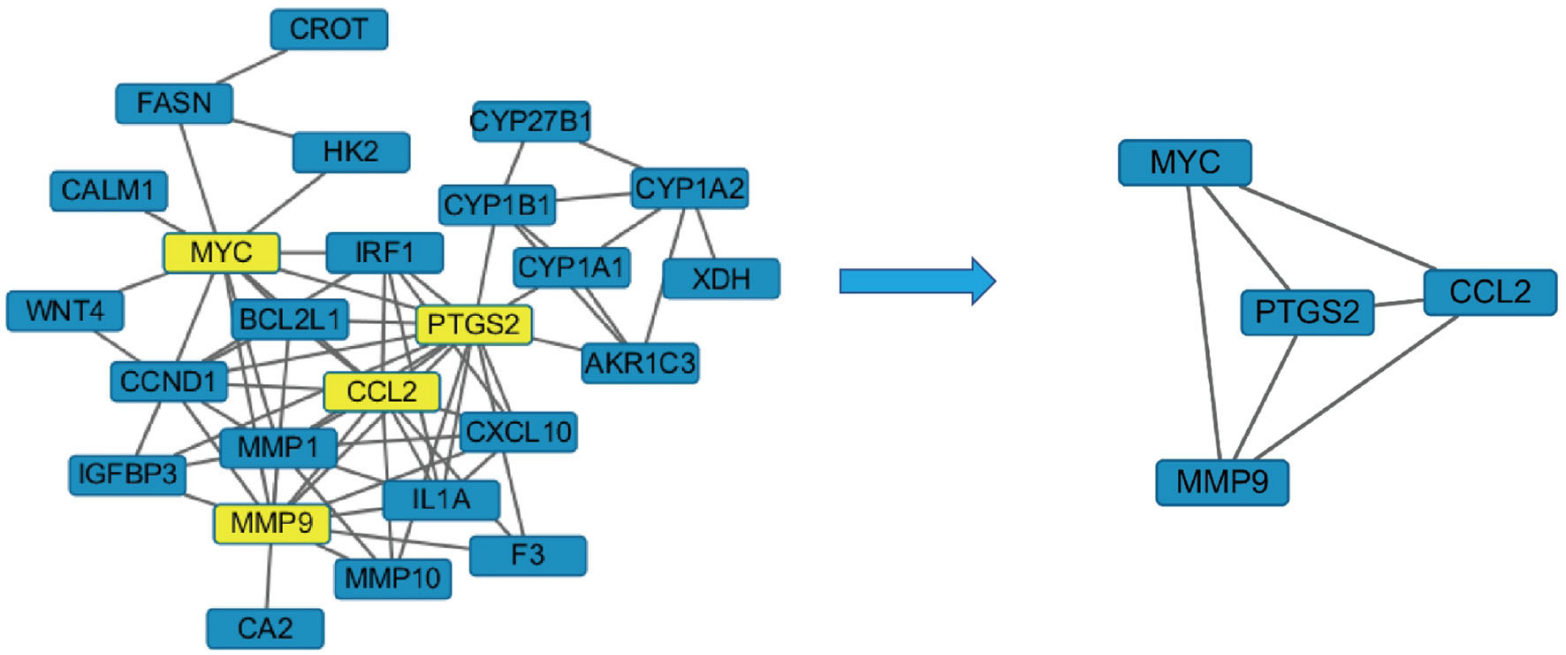
The disease target-PPI network.

The crossed target protein genes were uploaded to the STRING platform, and according to the highest screening condition of “highest confidence (≥0.9)”, a closely connected PPI network diagram was obtained. The network map was inputted into Cytoscape 3.2.1 software and screened with twice the average degree of freedom value to obtain four target proteins, including PTGS2, CCL2, MMP9, and MYC (**Figure 6**).

#### GO and KEGG Enrichment Analysis

**Figure 7:**
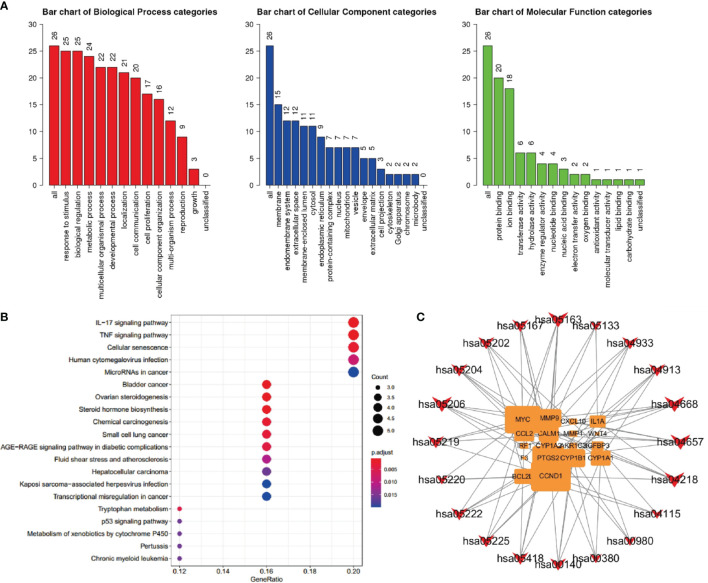
Component target network analysis of QYSLS in the treatment of EGFRI-related adverse skin reactions. **(A)** GO analysis of biological process, cellular component, and molecular function terms was performed on major targets of QYSLS. **(B)** KEGG analysis for the major targets of QYSLS. **(C)** Target-pathway network diagram of KEGG enrichment.

Bioconductor was used to upload 26 core target protein genes and conduct enrichment analysis of GO biological processes and KEGG signaling pathways. Thus, the potential mechanism of QYSLSL in the treatment of EGFRI-related adverse skin reactions was revealed. According to the results of enrichment analysis, the biological processes, cellular component, and molecular function involved mainly included the response to stimulus, membrane, protein binding, and other biological processes (**Figure 7A**). Similarly, the first 20 enriched KEGG signaling pathways were screened according to the significance of the p-value (see **Table 2** for details), which mainly involved the IL-17 signaling pathway and TNF signaling pathway. In **Figure 7B**, the size of bubbles represents the number of genes in this pathway, and the color represents the enrichment significance. **Figure 7C** shows the target-pathway network diagram, which describes the targets involved in each pathway in detail.

**Table 2:** Major targets in the KEGG pathway that are significantly enriched.

ID	Description	BgRatio	pvalue	p.adjust	qvalue	geneID	Count
hsa05219	Bladder cancer	41/8017	6.89E-06	0.000720553	0.000499484	MMP1/CCND1/MMP9/MYC	4
hsa04657	IL-17 signaling pathway	94/8017	8.79E-06	0.000720553	0.000499484	PTGS2/MMP1/MMP9/CCL2/CXCL10	5
hsa04913	Ovarian steroidogenesis	51/8017	1.67E-05	0.000847981	0.000587817	PTGS2/CYP1A1/CYP1B1/AKR1C3	4
hsa04668	TNF signaling pathway	112/8017	2.07E-05	0.000847981	0.000587817	PTGS2/MMP9/CCL2/CXCL10/IRF1	5
hsa00140	Steroid hormone biosynthesis	60/8017	3.19E-05	0.001045883	0.000725002	CYP1A2/CYP1A1/CYP1B1/AKR1C3	4
hsa05204	Chemical carcinogenesis	82/8017	0.000109206	0.002679772	0.001857609	PTGS2/CYP1A2/CYP1A1/CYP1B1	4
hsa04218	Cellular senescence	160/8017	0.000114381	0.002679772	0.001857609	CALM1/CCND1/MYC/IL1A/IGFBP3	5
hsa05222	Small cell lung cancer	92/8017	0.000170831	0.003502042	0.002427603	PTGS2/CCND1/BCL2L1/MYC	4
hsa04933	AGE-RAGE signaling pathway in diabetic complications	100/8017	0.000235742	0.004295752	0.0029778	CCND1/F3/CCL2/IL1A	4
hsa00380	Tryptophan metabolism	42/8017	0.000283835	0.004654888	0.003226751	CYP1A2/CYP1A1/CYP1B1	3
hsa05163	Human cytomegalovirus infection	225/8017	0.000558944	0.008333344	0.005776644	PTGS2/CALM1/CCND1/MYC/CCL2	5
hsa05418	Fluid shear stress and atherosclerosis	139/8017	0.000824819	0.01127252	0.00781407	CALM1/MMP9/CCL2/IL1A	4
hsa04115	p53 signaling pathway	72/8017	0.001386332	0.016096794	0.01115824	CCND1/BCL2L1/IGFBP3	3
hsa00980	Metabolism of xenobiotics by cytochrome P450	76/8017	0.001620746	0.016096794	0.01115824	CYP1A2/CYP1A1/CYP1B1	3
hsa05133	Pertussis	76/8017	0.001620746	0.016096794	0.01115824	CALM1/IL1A/IRF1	3
hsa05220	Chronic myeloid leukemia	76/8017	0.001620746	0.016096794	0.01115824	CCND1/BCL2L1/MYC	3
hsa05225	Hepatocellular carcinoma	168/8017	0.00166857	0.016096794	0.01115824	CCND1/BCL2L1/MYC/WNT4	4
hsa05206	MicroRNAs in cancer	310/8017	0.002349067	0.019863565	0.013769352	PTGS2/CYP1B1/CCND1/MMP9/MYC	5
hsa05167	Kaposi sarcoma-associated herpesvirus infection	186/8017	0.002422386	0.019863565	0.013769352	PTGS2/CALM1/CCND1/MYC	4
hsa05202	Transcriptional misregulation in cancer	186/8017	0.002422386	0.019863565	0.013769352	BCL2L1/MMP9/MYC/IGFBP3	4
hsa05323	Rheumatoid arthritis	93/8017	0.002889058	0.022562168	0.015640013	MMP1/CCL2/IL1A	3
hsa05205	Proteoglycans in cancer	204/8017	0.00338474	0.025231702	0.017490525	CCND1/MMP9/MYC/WNT4	4
hsa04625	C-type lectin receptor signaling pathway	104/8017	0.003963777	0.028263454	0.019592125	PTGS2/CALM1/IRF1	3
hsa04919	Thyroid hormone signaling pathway	119/8017	0.005779505	0.038152748	0.026447348	CCND1/MYC/WNT4	3
hsa05216	Thyroid cancer	37/8017	0.005815968	0.038152748	0.026447348	CCND1/MYC	2

#### Molecular Docking Results

**Figure 8:**
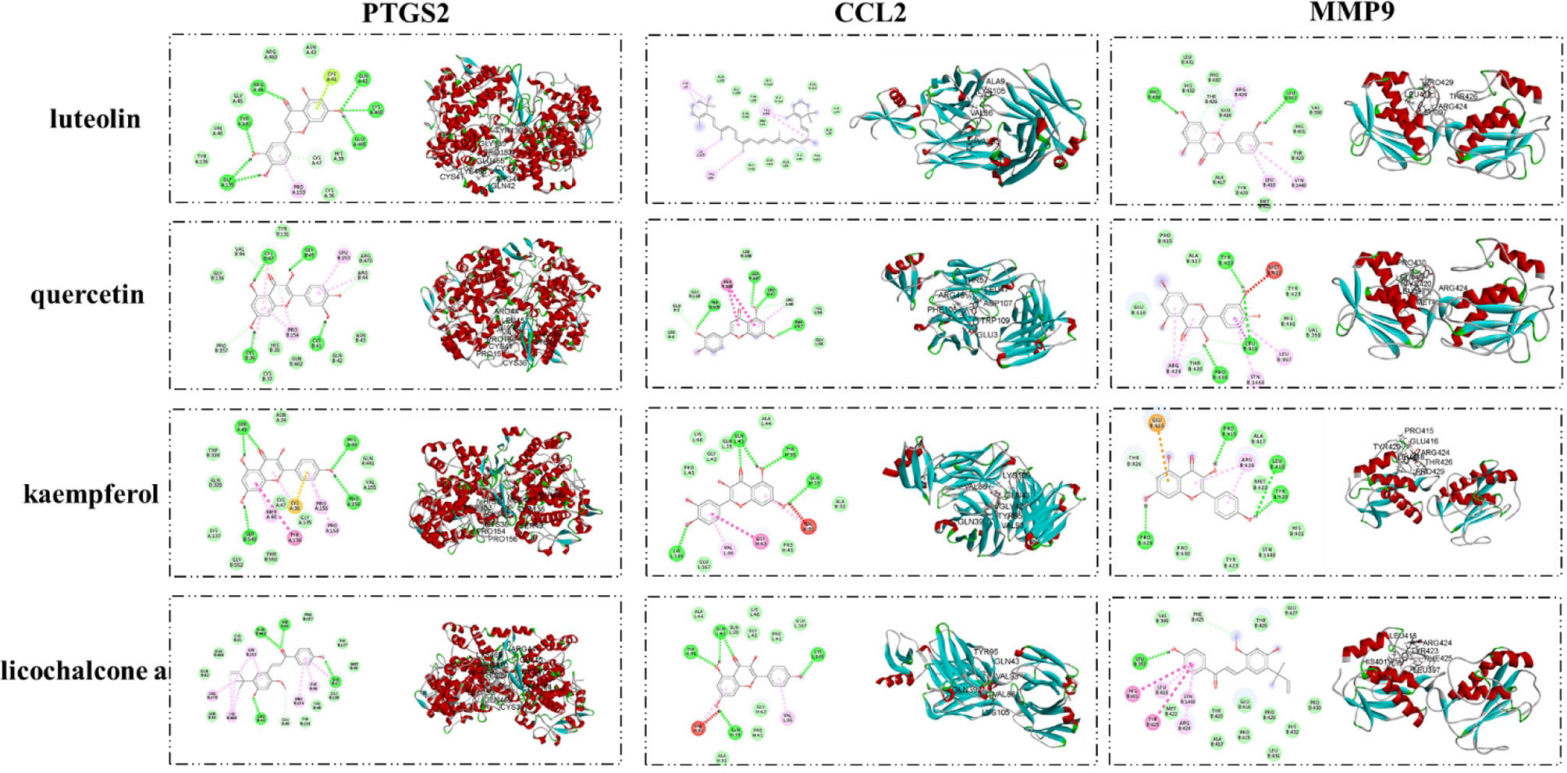
Validation of interactions between QYSLS compounds and targets.

The interaction between important active compounds and main targets was further studied by molecular docking. The binding affinity was less than −5.0 kcal/mol, indicating that the product had a good interaction. The molecular docking results showed that the active compounds luteolin, quercetin, kaempferol, and licochalcone a had good binding effects and reliable interactions with the conformation of the main protein targets CCL2, MMP9, and PTGS2. The conformation of key active compounds and major hub targets is shown in **Figure 8**. The binding affinity results are shown in **Table 3**.

**Table 3:** Virtual docking of four important active compounds in QYSLS to adverse skin reaction targets.

Ingredients	Binding energy		
	PTGS2	CCL2	MMP9
Luteolin	-7.06	-5	-5.86
Quercetin	-7.07	-5.43	-5.99
Kaempferol	-7	-6.16	-6.83
Licochalcone a	-7.36	-4.98	-6.87

### *In Vitro* Experiments

#### Effects of QYSLS on HaCaT Cell Proliferation

**Figure 9:**
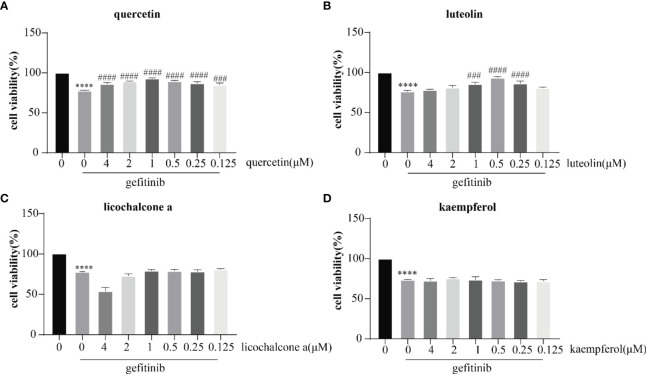
Inhibitory effects of quercetin **(A)** luteolin **(B)** licochalcone a **(C),** and kaempferol **(D)** on the proliferation of HaCaT cells. The drug concentration–cell viability curve was generated on the basis of cell viability data. All data were expressed as the mean ± SD. “*” compared with Control (The first pillar), ****p < 0.0001. “^#^” compared with Gefitinib (The second pillar), ^###^p < 0.005, ^####^p < 0.0001.

Cell damage is the main feature of adverse skin reactions caused by abnormal EGFR inhibition. In our analysis, several candidate targets were also involved in the regulation of cell damage. First, we examined the effects of gefitinib, luteolin, quercetin, licochalcone a, and kaempferol on the proliferation of human keratinocyte HaCaT cells *in vitro*. As shown in **Figures 9A–D**, different concentrations of luteolin and quercetin reversed the inhibitory effect of gefitinib on HaCaT cell proliferation.

#### Effects of QYSLS on the mRNA Expression of PTGS2, MMP9, and CCL2 in HaCaT Cells

**Figure 10:**
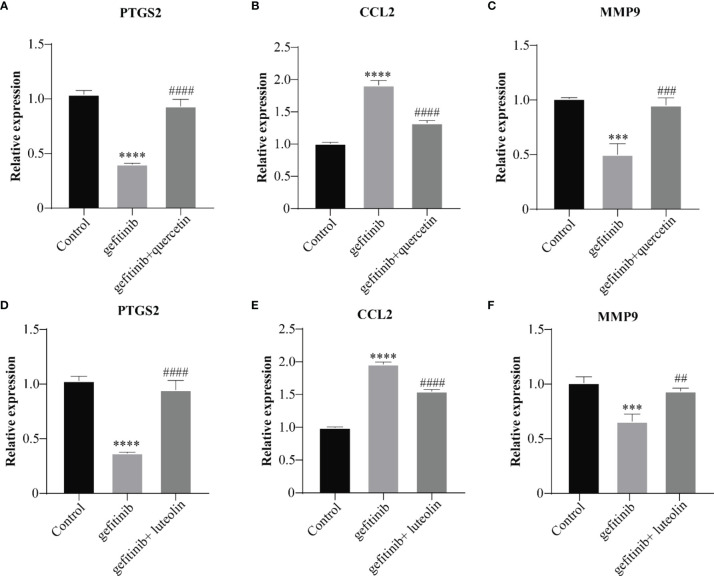
QYSLS mitigated EGFRI-induced adverse skin reactions by regulating the IL-17 and TNF pathway. qPCR was used to detect the mRNA expression levels of PTGS2, CCL2, and MMP9 in mice treated with quercetin **(A–C)** and luteolin **(D–F)**. “*”compared with Control, ***p < 0.005, ****p < 0.0001. “^#^”compared with Gefitinib, ^##^p < 0.01, ^###^p < 0.005, ^####^p < 0.0001.

The mRNA expression levels of PTGS2, MMP9, and CCL2 in the IL-17 and TNF pathway were verified by qPCR. Luteolin and quercetin decreased the levels of PTGS2 and MMP9 and increased the levels of CCL2. The results are shown in **Figures 10A–F**. Luteolin and quercetin in QYSLS were detected by HPLC (**Supplementary Figures S1**).

## Discussion

### Theoretical Basis of QYSLS in the Treatment of EGFRI-Related Adverse Skin Reactions

With the wide application of EGFRIs in the anti-tumor field, EGFRI-associated adverse reactions have attracted increasing attention, especially the high incidence of skin side effects. For example, the incidence of skin rash in lung cancer patients treated with EGFRIs is 60.2%. Currently, the internationally accepted treatment regimen for EGFRI-related adverse skin reactions is unsatisfactory. Therefore, TCM is considered to be a safe and effective alternative therapy in the treatment of EGFRI-related adverse skin reactions. As a folk prescription, QYSLS has been widely used in clinical settings. QYSLS is mainly used for the treatment of acne-like rashes caused by EGFRIs, with an effective rate of 90% (18). EGFRIs have been clinically applied for a long time with reliable efficacy. In addition, we achieved good efficacy in a rat model of gefitinib-induced adverse skin reactions with QYSLS (19). To further study the efficacy and potential mechanism of QYSLS, several *in vitro* and *in vivo* experiments were conducted in this study. Combined network pharmacology and molecular docking biological approaches were used to confirm its effectiveness and molecular mechanisms.

### Component Analysis of QYSLS in the Treatment of EGFRI-Related Adverse Skin Reactions

In this study, luteolin and quercetin were found to be the main active ingredients involved in the treatment of EGFRI-related adverse skin reactions. Luteolin is one of the most abundant secondary metabolites in several medicinal plants, and it has various pharmacological activities. As a natural flavonoid, luteolin (3,4,5,7-tetrahydroxy flavonoids) exists in a variety of vegetables, fruits, and medicinal plants, including broccoli, onion leaves, carrots, peppers, cabbage, apple peels, and chrysanthemums (20). Luteolin has been reported to have antioxidant, antimicrobial, anti-inflammatory, chemoprophylaxis, chemotherapy, cardioprotective, antidiabetic, neuroprotective, and anti-allergic properties (21). Luteolin shows great potential to inhibit and even reverse skin diseases (such as psoriasis and dermatitis) and ultra-violet-induced diseases (such as skin cancer and photoaging) (22). A variety of pharmacological activities of luteolin have been confirmed to be related to its anti-inflammatory effect (23). The anti-inflammatory activity of luteolin includes the inhibition of pro-inflammatory mediators (e.g., COX-2, NO, IL-6, IL 1β, TNF-α) and regulation of multiple signaling pathways, including NF-κB, AP-1, and JAK-STAT (24).

Quercetin (chemical formula C15H10O7) is a polyphenolic flavonoid widely found in nature in a variety of vegetables and fruits, such as apples, red grapes, onions, raspberries, honey, cherries, citrus fruits, and green leafy vegetables (25). The regulation of inflammation is one of the core and most significant effects of quercetin. Quercetin has been shown to have anti-inflammatory activities in several *in vivo* and *in vitro* studies by inhibiting inflammatory cytokines and enzymes (26). Quercetin regulates IL-1α, IL-1β, IL-2, IL-10, MCP-1, COX2, MMP-1, and SOCS, playing an anti-inflammatory role (27). Atopic dermatitis is a prevalent inflammatory skin disease worldwide, and recent studies found that quercetin regulates several pathways to treat this skin disease (28). In conclusion, both luteolin and quercetin effectively treat skin diseases through their anti-inflammatory effects.

Quercetin comes from licorice, *Astragalus membranaceus*, and *Lonicera japonica*. Luteolin comes from *Lonicera japonica*. It can be seen that *Lonicera japonica* has the most anti-inflammatory effect, followed by licorice and *Astragalus*. The efficacy of individual drugs will be further studied in future experiments.

### Target Analysis of QYSLS in the Treatment of EGFRI-Related Adverse Skin Reactions

PTGS2, also known as cyclooxygenase 2 (COX2), is a subtype of prostaglandin-endoperoxide synthase. Prostaglandins are induced by diverse factors to produce and release prostaglandins and regulate inflammatory responses. They exist in several tissues and are expressed by a large number of immune cells, such as macrophages, at inflammatory sites. PTGS2 is widely used as an inflammatory marker of skin inflammation and is related to the pathogenesis of skin injury (29, 30). The matrix metalloproteinase MMP9 regulates CXCL8 and VEGFA and participates in inflammatory responses (31). MMP9 has been shown to be involved in skin wound healing (32). CCL2, also known as monocyte chemotactic protein 1 (MCP-1), was one of the first chemokines to be discovered and has been found to have a strong chemotactic ability to recruit monocytes and macrophages (33). It has been reported that EGFR inhibition increases IL-1 signaling by reducing IL-1R2 and upregulating CCL2 and CCL5, thereby leading to the infiltration of skin neutrophils (34). CCL2 is also upregulated in EGFR^△EP^ transgenic mice (35). Therefore, PTGS2, MMP9, and CCL2 are target proteins related to inflammation and the pathogenesis of skin diseases.

### Pathway Mechanism Analysis of QYSLS in the Treatment of EGFRI-Related Adverse Skin Reactions

In this study, KEGG pathway enrichment revealed the IL-17 and TNF signaling pathway as the most relevant pathway. It was reported that IL-17 was upregulated in mouse skin after EGFRI treatment with erlotinib, suggesting that IL-17A is involved in the mechanism of EGFRI-related adverse skin reactions (35). In our previous protein chip detection in a rat adverse skin reaction model, the IL-17 signaling pathway was also enriched (19). The IL-17 family is a subset of cytokines composed of IL-17A–F that play a critical role in both acute and chronic inflammatory responses. IL-17A is a marker of the T-helper cell 17 subpopulation, which protects the host from extracellular pathogens and participates in inflammatory responses in autoimmune diseases (36). The IL-17 signaling pathway is a key signaling pathway in the inflammatory response of skin T cells, B cells, and macrophages. In addition, IL-17A signaling is an important pathogenic mechanism of psoriasis. The main target cells of the IL-17A-related signaling pathway include epithelial cells, keratinocytes, macrophages, T/B cells, and fibroblasts, which activate downstream pathways mainly through the interaction of receptors and ligands. In addition to triggering skin inflammation, IL-17A stimulates the proliferation of keratinocytes, which also produce a variety of antimicrobial peptides and chemokines (37). TNFα is a cytokine that can directly kill tumor cells and has no obvious cytotoxicity to normal cells. It participates in systemic inflammatory response and is one of the cytokines that constitute the acute phase response, mainly produced by activated macrophages (38). It has been reported that TNFa and IL-1 are involved in EGFRIs-related skin inflammatory reactions in mouse models. TNF-α inhibitors can reduce skin inflammation caused by EGFRIs (39). Therefore, both IL-17 and TNF signaling pathways are involved in the pathogenesis of skin inflammation caused by EGFRIs. By further exploring the connection between TNF signaling pathway and IL-17 signaling pathway, we found that TNF is not only an effector but also a promoter of inflammatory Th differentiation, thus promoting the production of inflammatory cytokines such as IL-17 (40). How QYSLS plays a role through these two pathways needs further research.

## Conclusion

QYSLS may play an anti-inflammatory role by acting on the target proteins PTGS2, MMP9 and CCL2 related to the IL-17 and TNF signaling pathway, thereby treating the adverse skin reactions caused by EGFR inhibition. The active components of QYSLS are luteolin and quercetin.

## Data Availability Statement

The original contributions presented in the study are included in the article/**Supplementary Material**. Further inquiries can be directed to the corresponding authors.

## Ethics Statement

The animal study was reviewed and approved by the Animal Ethics Committee of Beijing University of Chinese Medicine.

## Author Contributions

YW participated in the study design and the first draft and was responsible for the whole experiment. YW, YZ, and CD are responsible for revising the article. YZ, CD, CJ, HZ, TP, SC, WC, and XW contributed to the data analysis. All authors conducted some experiments and analyzed the data. YT, XW, ZL, and PW offered to help revise the manuscript. MJ and QH conceptualized and designed the study. All authors contributed to the article and approved the submitted version.

## Funding

This study was supported by grants of the National Natural Science Foundation of China (Grant Nos. 81973667, 81973690).

## Conflict of Interest

The authors declare that the research was conducted in the absence of any commercial or financial relationships that could be construed as a potential conflict of interest.

## Publisher’s Note

All claims expressed in this article are solely those of the authors and do not necessarily represent those of their affiliated organizations, or those of the publisher, the editors and the reviewers. Any product that may be evaluated in this article, or claim that may be made by its manufacturer, is not guaranteed or endorsed by the publisher.
