# Supplementary figures and images for: Exploration of the Potential Mechanism of Qi Yin San Liang San Decoction in the Treatment of EGFRI-Related Adverse Skin Reactions Using Network Pharmacology and In Vitro Experiments
Source: Front Oncol. 2022 Mar 15;12:790713. doi: 10.3389/fonc.2022.790713 (PMC8964498; doi:10.3389/fonc.2022.790713)

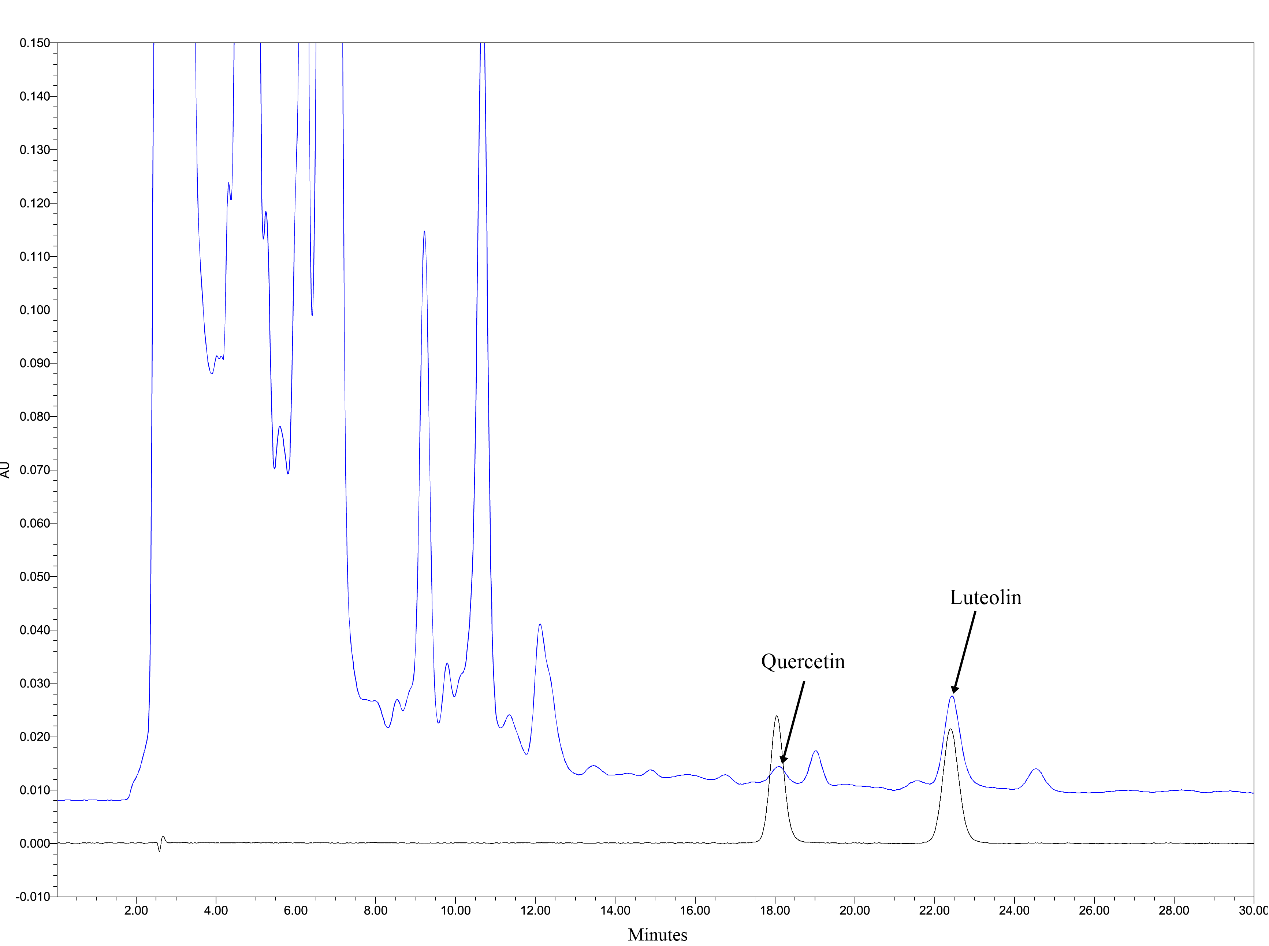


**Supplementary Figure 1.** Component Validation of luteolin and quercetin in QYSLS by HPLC.

Supplement: Supplementary file 1 [file Image_1.docx]
